# High-throughput sequencing analysis of the rhizosphere arbuscular mycorrhizal fungi (AMF) community composition associated with *Ferula sinkiangensis*

**DOI:** 10.1186/s12866-020-02024-x

**Published:** 2020-11-03

**Authors:** Yunfeng Luo, Zhongke Wang, Yaling He, Guifang Li, Xinhua Lv, Li Zhuang

**Affiliations:** College of life Sciences, Shihezi University, Shihezi City, 832003 Xinjiang China

**Keywords:** Arbuscular mycorrhizal fungi (AMF), *Ferula sinkiangensis*, Illumina MiSeq, Diversity, Community composition, Soil physiochemical

## Abstract

**Background:**

*Ferula sinkiangensis* is an increasingly endangered medicinal plant. Arbuscular mycorrhiza fungi (AMF) are symbiotic microorganisms that live in the soil wherein they enhance nutrient uptake, stress resistance, and pathogen defense in host plants. While such AMF have the potential to contribute to the cultivation of *Ferula sinkiangensis*, the composition of AMF communities associated with *Ferula sinkiangensis* and the relationship between these fungi and other pertinent abiotic factors still remains to be clarified.

**Results:**

Herein, we collected rhizosphere and surrounding soil samples at a range of depths (0–20, 20–40, and 40–60 cm) and a range of slope positions (bottom, middle, top). These samples were then subjected to analyses of soil physicochemical properties and high-throughput sequencing (Illumina MiSeq). We determined that *Glomus* and *Diversispora* species were highly enriched in all samples. We further found that AMF diversity and richness varied significantly as a function of slope position, with this variation primarily being tied to differences in relative *Glomus* and *Diversispora* abundance. In contrast, no significant relationship was observed between soil depth and overall AMF composition, although some AMF species were found to be sensitive to soil depth. Many factors significantly affected AMF community composition, including organic matter content, total nitrogen, total potassium, ammonium nitrogen, nitrate nitrogen, available potassium, total dissolvable salt levels, pH, soil water content, and slope position. We further determined that Shannon diversity index values in these communities were positively correlated with total phosphorus, nitrate-nitrogen levels, and pH values *(P* < 0.05), whereas total phosphorus, total dissolvable salt levels, and pH were positively correlated with Chao1 values (*P* < 0.05).

**Conclusion:**

In summary, our data revealed that *Glomus* and *Diversispora* are key AMF genera found within *Ferula sinkiangensis* rhizosphere soil. These fungi are closely associated with specific environmental and soil physicochemical properties, and these soil sample properties also differed significantly as a function of slope position (*P* < 0.05). Together, our results provide new insights regarding the relationship between AMF species and *Ferula sinkiangensis*, offering a theoretical basis for further studies of their development.

**Supplementary Information:**

The online version contains supplementary material available at 10.1186/s12866-020-02024-x.

## Background

*Ferula sinkiangensis* is a perennial plant found only in the Yining region of Xinjiang province, China that blooms only once, and that produces seedlings each March [1, 2]. In years when these plants do not reach the flowering stage, their root systems instead gradually expand and tufted basal leaves develop. After entering the flowering stage, these plants bloom at the beginning of May, bear fruit by mid-late May, and die at the end of June [3, 4]. *Ferula sinkiangensis* exhibits potent anticancer [5], antibiotic [6], antiviral [7], antioxidant [8], and anti-inflammatory [9] properties, and was thus included in the Pharmacopoeia of the People’s Republic of China in 1977 [4]. Extensive *Ferula sinkiangensis* harvesting in recent years, however, has caused serious habitat damage [10]. This, coupled with its low reproductive rate and the prevalence of pests, diseases, and poor environmental conditions, has led *Ferula sinkiangensis* to become increasingly endangered [11]. Efforts to conserve this valuable medicinal herb are thus essential.

Slope position is an important topographical factor that governs microenvironmental heterogeneity by impacting the temperature, light, soil physicochemical properties, and water levels to which plants are exposed [12, 13]. Abiotic and biotic factors additionally vary with soil depth [14]. Soil nutrient content and environmental gradients observed as a function of depth may influence the abundance, composition, and function of soil microbial communities [15]. Although slope position and depth are not direct ecological factors that govern microbial survival, they can influence microbe distributions by controlling the spatiotemporal distribution of a range of ecological factors and combinations thereof. However, the impacts of slope position and soil depth on the rhizosphere arbuscular mycorrhizal fungi (AMF) community composition associated with *Ferula sinkiangensis* remain poorly understood.

Soil microorganisms have been an increasingly important focus of ecological research [16], as they have been shown to be key regulators of plant growth, development, and overall ecosystem stability [17]. AMF species represent a particularly important subset of soil microbes that are able to form mutualistic symbiotic relationships with the roots of > 80% of all land plants [18–20]. Mycorrhizal symbionts are key sources of plant nutrients, and these AMF species can also enhance host plant resistance to environmental stressors [21, 22] and to pathogen infections [23, 24]. Few studies to date, however, have conducted comprehensive evaluations of AMF in the rhizosphere soil associated with *Ferula sinkiangensis*, and current understanding of the diversity and distribution of these microbial communities remains limited. In addition, the association between these microbes and soil physicochemical properties remains uncertain. As such, it is important that field studies of these AMF communities be conducted.

AMF exhibit rich species diversity, and approaches to studying these fungi have historically included both spore identification efforts and molecular analyses [25, 26]. Spore identification, however, is a time- and energy-intensive task that is susceptible to variability in spore morphology as a function of regional variations, host species, and microbial age, making it challenging to differentiate between the spores of similar species [27]. In contrast, high-throughput sequencing [28, 29] represents an increasingly robust and common approach to reliably studying AMF community structure and diversity. High-throughput approaches have been widely used in studies of AMF in the context of forestry [13], agriculture [30, 31], and environmental remediation [32], while they have also been used to explore the relationship between slope position and soil depth on AMF community composition associated with specific plants.

While prior studies have leveraged high-throughput sequencing to evaluate rhizosphere microorganisms associated with *Ferula sinkiangensis* [33, 34], no studies have comprehensively assessed the diversity, community composition, or distribution patterns of AMF species associated with this plant. As such, in the present study, we employed an Illumina MiSeq sequencing approach [29] to assess the diversity and structure of rhizosphere AMF communities associated with *Ferula sinkiangensis.* The goals of this study were as follows: (1) to research the rhizosphere AMF diversity associated with *Ferula sinkiangensis*; (2) to evaluate AMF community composition and distribution patterns as a function of slope position and soil depth; and (3) to discover the important topographic and edaphic factors affecting AMF diversity, community composition, and distribution patterns.

## Results

### AMF species diversity

We identified 77 total AMF OTUs in our 27 soil samples, which were separated into 9 groups. Dilution curves generated for these 9 groups were flat, indicating that sequencing depth was sufficient and that additional sequencing depth would have revealed only a small number of additional species (Supplementary Fig. S1).

We next conducted taxonomic analyses of these representative OTUs, leading us to determine that these fungi were associated with 1 Class, 4 Orders, 4 Families, 4 Genera, and 20 Species, with additional unidentified species having been detected at various taxonomic levels. No significant differences in Alpha diversity, Sobs, Shannon, Chao1, or Simpson index values were observed among samples as a function of soil depth, whereas these index values did vary significantly as a function of slope position. Phylogenetic diversity (PD) was unrelated to soil depth or slope position. Greater than 99.98% coverage was achieved in this sequencing analysis, confirming that these data met with the targeted sequencing depth requirements (Table 1).

**Table 1:** Rhizosphere AMF diversity indices

depth	sobs	shannon	simpson	chao	pd	coverage
sw						
0-20 cm	20.67 ± 6.03	2.09 ± 0.35	0.21 ± 0.09	21.00 ± 5.57	1.43 ± 0.52	99.99%
20-40 cm	28.00 ± 1.73	2.27 ± 0.15	0.15 ± 0.02	28.87 ± 1.86	1.97 ± 0.33	99.98%
40-60 cm	23.67 ± 6.66	2.04 ± 0.33	0.23 ± 0.08	25.00 ± 5.68	1.78 ± 0.50	99.98%
mean	24.11 ± 5.58**B**	2.13 ± 0.27**B**	0.20 ± 0.07**B**	24.96 ± 5.31**B**	1.72 ± 0.46	99.99%
zw						
0-20 cm	16.00 ± 7.00	1.51 ± 0.26	0.34 ± 0.08	116.33 ± 7.02	1.45 ± 0.45	99.99%
20-40 cm	17.00 ± 5.57	1.51 ± 0.22	0.34 ± 0.15	17.50 ± 6.06	1.56 ± 0.74	99.99%
40-60 cm	20.00 ± 2.00	1.75 ± 0.45	0.25 ± 0.11	21.00 ± 2.65	2.14 ± 0.47	99.98%
mean	15.67 ± 4.92**C**	1.60 ± 0.31**C**	0.31 ± 0.11**A**	18.28 ± 5.26**C**	1.72 ± 0.59	99.99%
xw						
0-20 cm	30.00 ± 6.25	2.41 ± 0.16	0.12 ± 0.04	35.33 ± 13.65	1.45 ± 0.22	99.98%
20-40 cm	29.33 ± 1.15	2.35 ± 0.05	0.15 ± 0.03	29.67 ± 1.53	1.53 ± 0.05	99.99%
40-60 cm	31.33 ± 1.15	2.48 ± 0.09	0.13 ± 0.03	33.33 ± 4.16	1.62 ± 0.21	99.99%
mean	30.22 ± 3.35**A**	2.41 ± 0.11**A**	0.13 ± 0.03**C**	32.75 ± 7.60**A**	1.54 ± 0.17	99.99%

Lowercase letters are significantly difference among three soil depth,capital letters are significantly difference among three plant species (*P* < 0.05). SW, ZW, and XW respectively correspond to the top, middle, and bottom slope positions

**Fig. 1:**
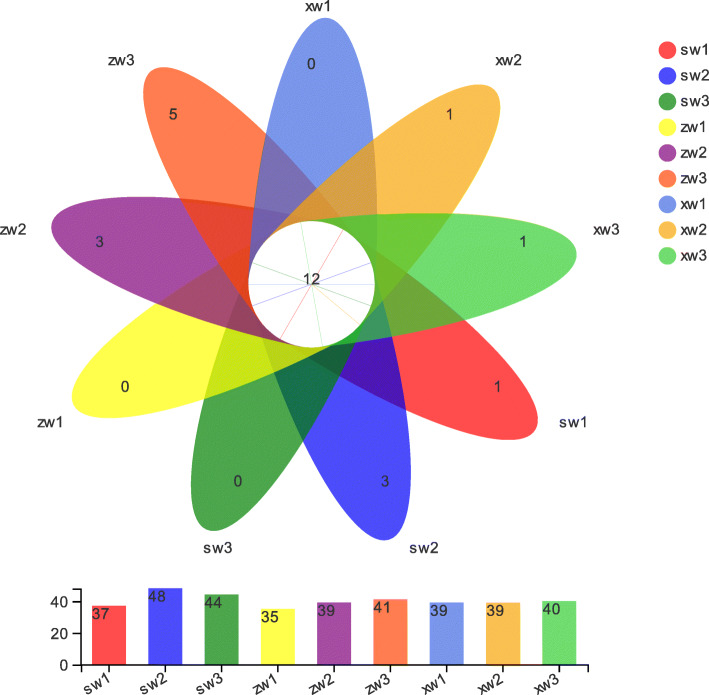
Operational taxonomic units (OTU)-based petal maps. Each petal corresponds to a sample group, with the shared overlapping region representing OTUs common to all samples, and the numbers on individual petals representing the number of OTUs unique to a given sample group. SW, ZW, and XW respectively correspond to the top, middle, and bottom slope positions. 1, 2, and 3 respectively represent samples collected at a soil depth of 0-20 cm, 20-40 cm, and 40-60 cm

Intermediate junctions were used to identify OTUs common to all samples as well as OTUs unique to specific samples. These analyses revealed that there were 12 core OTUs in our samples, with two of these OTUs belonging to the *Diversispora* genus and all others belonging to the *Glomus* genus (Fig. 1).

### AMF community composition and distribution

**Fig. 2:**
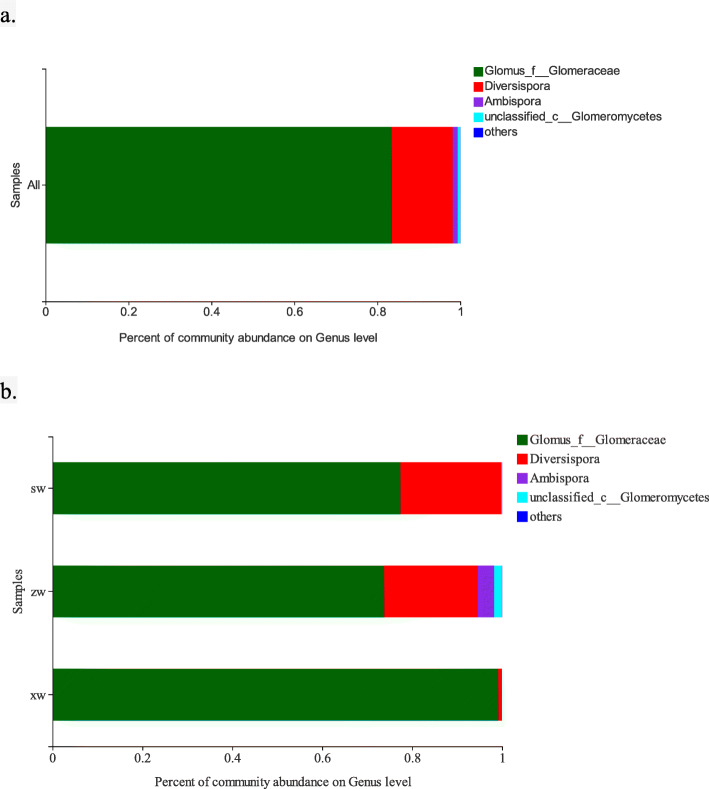
The proportion of AMF genera in soil. All the soil samples (**a**) and soil samples at different slope positions (**b**). SW, ZW, and XW respectively correspond to the top, middle, and bottom slope positions

Clear differences in AMF community composition in different rhizosphere soil samples were observed in this study. *Glomus* species accounted for 83.46% of total AMF species, while *Diversispora* accounted for 14.73%, *Ambispora* for 1.21%, and *Paraglomus* species were only detected in a few samples (Fig. 2a).

In our constructed species relationship diagrams, *Glomus* distributions in individual samples ranged from 7.7–13%. Of these *Glomus* species, 31, 28, and 39% were detected in the soil samples from the upper, middle, and lower slope positions, respectively, while 31, 28, and 39% of *Glomus* species were detected in samples collected at respective soil depths of 0–20 cm, 20–40 cm, and 40–60 cm. *Diversispora* species were primarily distributed in the upper and middle slope positions, with 52, 47.8, and 0.2% of these species being found in the upper, middle, and lower slope positions, respectively. In addition, 30, 31, and 39% of *Diversispora* species were detected in samples collected at 0–20 cm, 20–40 cm, and 40–60 cm depths, respectively (Fig. 2b). *Glomus* and *Diversispora* species were present at all soil depths and slope positions, with *Glomus* species composing the largest proportion of all samples. *Ambispora* species were only detected in soil samples collected at depths of 0–20 cm and 40–60 cm from the middle slope position (Supplementary Fig. S2).

**Fig. 3:**
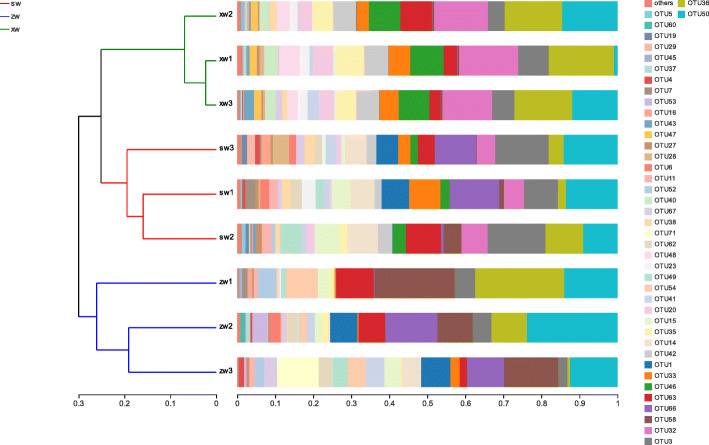
A UPGMA tree based on Unweighted Unifrac Distances at the OTU level. The UPGMA cluster tree structure is shown on the left, while on the right is the relative abundance distributions at the OTU level for each sample. SW, ZW, and XW respectively correspond to the top, middle, and bottom slope positions. 1, 2, and 3 respectively represent samples collected at a soil depth of 0-20 cm, 20-40 cm, and 40-60 cm

A cluster analysis conducted according to Unweighted UniFrac distance values revealed that samples were separable into three primary categories based upon environmental variables. Clusters of samples collected at the same slope site indicated that AMF community composition was more similar at a given slope site, whereas this composition differed substantially among slope sites (Fig. 3).

**Fig. 4:**
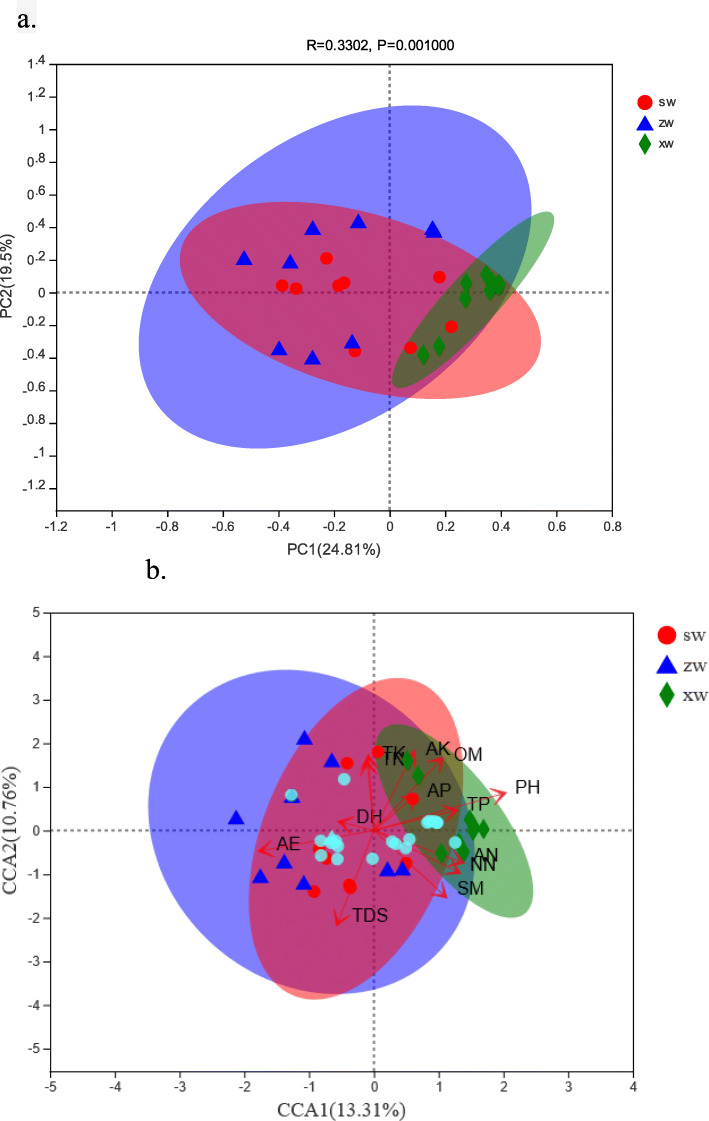
A Bray-Curtis PCoA analysis on OTU levels (**a**). CCA analysis based on OTU levels and sample slope positions (**b**). Blue triangles, red circles, and green diamonds correspond to samples collected at the middle (ZW), top (SW), and bottom (XW) of the slope, respectively. The light blue circles correspond to the top 20 OTUs by abundance

A Bray-Curtis PCoA analysis indicated that there was no difference in AMF community composition at different soil depths, whereas these communities did differ significantly as a function of sample slope position. While there were no differences in AMF community composition in the middle or upper slope positions, the composition of these samples did differ significantly with respect to the composition of AMF communities in soil samples collected from lower slope positions (*R* = 0.332, *P* = 0.001) (Fig. 4a).

**Fig. 5:**
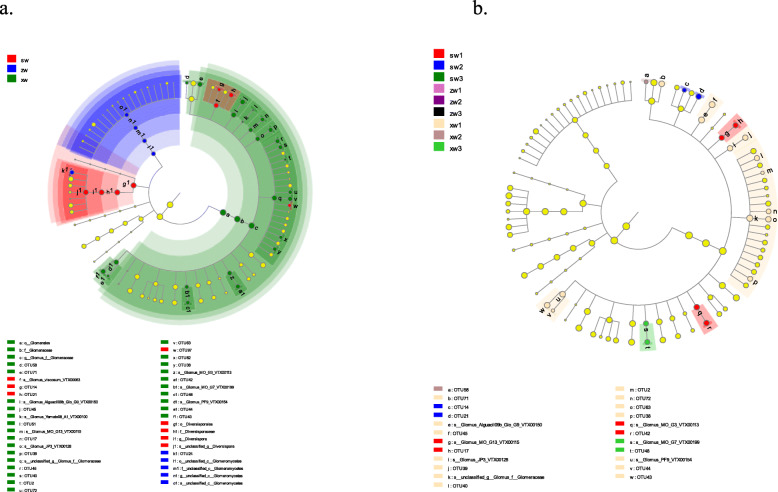
A linear discriminant analysis effect size (Lefse) analysis of differences in AMF community composition as a function of soil depth and slope position. Differently colored nodes correspond to microbial groups that were significantly enriched in the corresponding groups and that significantly influenced the differences between groups. Light yellow nodes correspond to microbial groups with no significant differences in different groups or that had no significant influence on the difference between groups. SW, ZW, and XW respectively correspond to the top, middle, and bottom slope positions. 1, 2, and 3 respectively represent samples collected at a soil depth of 0-20 cm, 20-40 cm, and 40-60 cm

LEFse analyses were also used to identify biomarkers associated with AMF diversity in rhizosphere soil. At different soil depths, only *Glomus* species were significantly enriched at the species and OTU levels. The most abundant biomarkers were detected in soil samples collected at a depth of 0–20 cm, with decreasing levels of these biomarkers as soil depth increased (Fig. 5b). With respect to slope position, biomarker abundance was highest in the lower slope position. However, *Glomus* species were significantly enriched in samples collected from the lower slope position, whereas *Diversispora* species were significantly enriched in samples collected from the upper slope position (Fig. 5a).

### Relationships between soil AMF diversity and soil properties

Significant differences in pH and TP were observed between soil samples as a function of soil depth (Table 2; *P* < 0.05), but these differences may be a function of different slope positions, as soil from the same slope position did not differ as a function of depth. When comparing soil samples collected from different slope positions, there were significant differences in OM, TN, TP, TK, AN, NN, TDS, pH, and SM values (*P* < 0.05).

**Table 2:** Mean values of nonbiological factors in soils of different depths

depth	OM(g/kg)	TN(g/kg)	TP(g/kg)	TK(g/kg)	NN (mg/kg)	AN (mg/kg)	AP (mg/kg)	AK (mg/kg)	TDS(g/kg)	PH	SM(%)
sw											
0 -20 cm	23.47 ± 3.75	1.99 ± 0.34	0.76 ± 0.09**a**	11.82 ± 2.94	3.71 ± 1.05	5.59 ± 0.62	3.05 ± 0.62	239.71 ± 99.13	5.70 ± 3.29	8.07 ± 0.14**b**	7.44 ± 1.73
20-40 cm	23.12 ± 1.88	2.00 ± 0.23	0.77 ± 0.09**a**	11.86 ± 2.95	3.37 ± 0.61	5.58 ± 0.99	4.32 ± 2.06	217.33 ± 66.7	5.45 ± 3.15	7.95 ± 0.15**b**	8.00 ± 1.24
40-60 cm	21.92 ± 1.42	2.03 ± 0.07	0.76 ± 0.07**a**	12.05 ± 2.42	3.72 ± 1.10	5.06 ± 1.24	3.11 ± 1.05	234.01 ± 82.87	5.55 ± 3.20	7.98 ± 0.15**b**	6.5 ± 1.65
mean	22.84 ± 2.32**B**	2.00 ± 0.21**B**	0.76 ± 0.07**A**	11.91 ± 2.41**B**	3.60 ± 0.84**B**	5.41 ± 0.89**AB**	3.49 ± 1.34	230.35 ± 73.40	6.13 ± 4.83**A**	8.00 ± 0.14**B**	7.31 ± 1.50**A**
zw											
0 -20 cm	24.13 ± 2.58	2.14 ± 0.21	0.53 ± 0.03**b**	15.08 ± 1.79	3.62 ± 2.36	4.40 ± 0.61	2.43 ± 0.68	200.11 ± 19.55	5.64 ± 3.26	8.04 ± 0.16**b**	5.38 ± 2.7
20-40 cm	23.52 ± 3.73	2.16 ± 0.18	0.54 ± 0.02**b**	15.60 ± 1.6	3.68 ± 1.74	4.80 ± 0.35	2.17 ± 0.56	253.91 ± 101.89	5.86 ± 3.38	8.03 ± 0.11**b**	5.54 ± 9.80
40-60 cm	25.40 ± 1.11	2.30 ± 0.02	0.55 ± 0.01**b**	16.48 ± 0.45	4.46 ± 1.64	4.22 ± 0.32	2.35 ± 0.50	251.63 ± 28.73	4.43 ± 2.55	8.19 ± 0.10**b**	5.20 ± 3.80
mean	24.35 ± 2.48A**B**	2.20 ± 0.16**A**	0.53 ± 0.21**C**	15.72 ± 1.37**A**	3.92 ± 1.73A**B**	4.47 ± 0.46**B**	2.31 ± 0.52	235.22 ± 59.93	5.20 ± 4.67**AB**	8.09 ± 0.13**B**	5.37 ± 0.56**B**
xw											
0 -20 cm	27.24 ± 1.79	2.16 ± 0.13	0.70 ± 0.02**a**	15.06 ± 1.47	5.56 ± 2.25	5.56 ± 1.24	3.09 ± 1.65	314.8 ± 257.37	0.28 ± 0.16	8.58 ± 0.10**a**	7.76 ± 1.66
20-40 cm	26.50 ± 2.04	2.16 ± 0.10	0.70 ± 0.01**a**	15.04 ± 1.57	5.08 ± 2.17	5.74 ± 0.47	3.57 ± 2.84	359.52 ± 284.25	0.45 ± 0.26	8.62 ± 0.11**a**	7.21 ± 3.08
40-60 cm	26.02 ± 2.99	2.18 ± 0.13	0.70 ± 0.05**a**	15.35 ± 1.6	5.59 ± 3.20	6.33 ± 0.78	3.17 ± 1.38	419.81 ± 342.26	0.45 ± 0.26	8.56 ± 0.23**a**	6.73 ± 1.81
mean	26.59 ± 2.09**A**	2.17 ± 0.10**A**	0.70 ± 0.03**B**	15.15 ± 1.35**A**	5.41 ± 2.25**A**	5.87 ± 0.84**A**	3.28 ± 1.80	364.71 ± 261.01	2.08 ± 0.49**B**	8.59 ± 0.14**A**	7.23 ± 2.02**A**

Lowercase letters are significantly difference among three soil depth, capital letters are significantly difference among three plant species (*P* < 0.05). SW, ZW, and XW respectively correspond to the top, middle, and bottom slope positions

Spearman correlation analyses revealed that TP, AN, and soil pH were significantly positively correlated with Shannon index values (*P* < 0.05), whereas TP, TDS, pH, AE, and Chao 1 values were positively correlated with one another (*P* < 0.05) (Supplementary Table S1).

CCA analysis revealed that OM (*R*^2^ = 0.3939, *P* = 0.002), TN (*R*^2^ = 0.2701, *P* = 0.021), TK (*R*^2^ = 0.3209, *P* = 0.012), NN (*R*^2^ = 0.3003, *P* = 0.012), AN (*R*^2^ = 0.2692, *P* = 0.03), AK (*R*^2^ = 0.3803, *P* = 0.001), TDS (*R*^2^ = 0.5328, *P* = 0.001), pH (*R*^2^ = 0.4834, *P* = 0.001), SM (*R*^2^ = 0.4108, *P* = 0.004), and AE (*R*^2^ = 0.335, *P* = 0.011) all had a significant impact on AMF community composition, explaining 24.07% of the overall variability in this composition. In addition, AE was positively correlated with TDS and depth, whereas it was negatively correlated with other environmental factors (Fig. 4b).

**Fig. 6:**
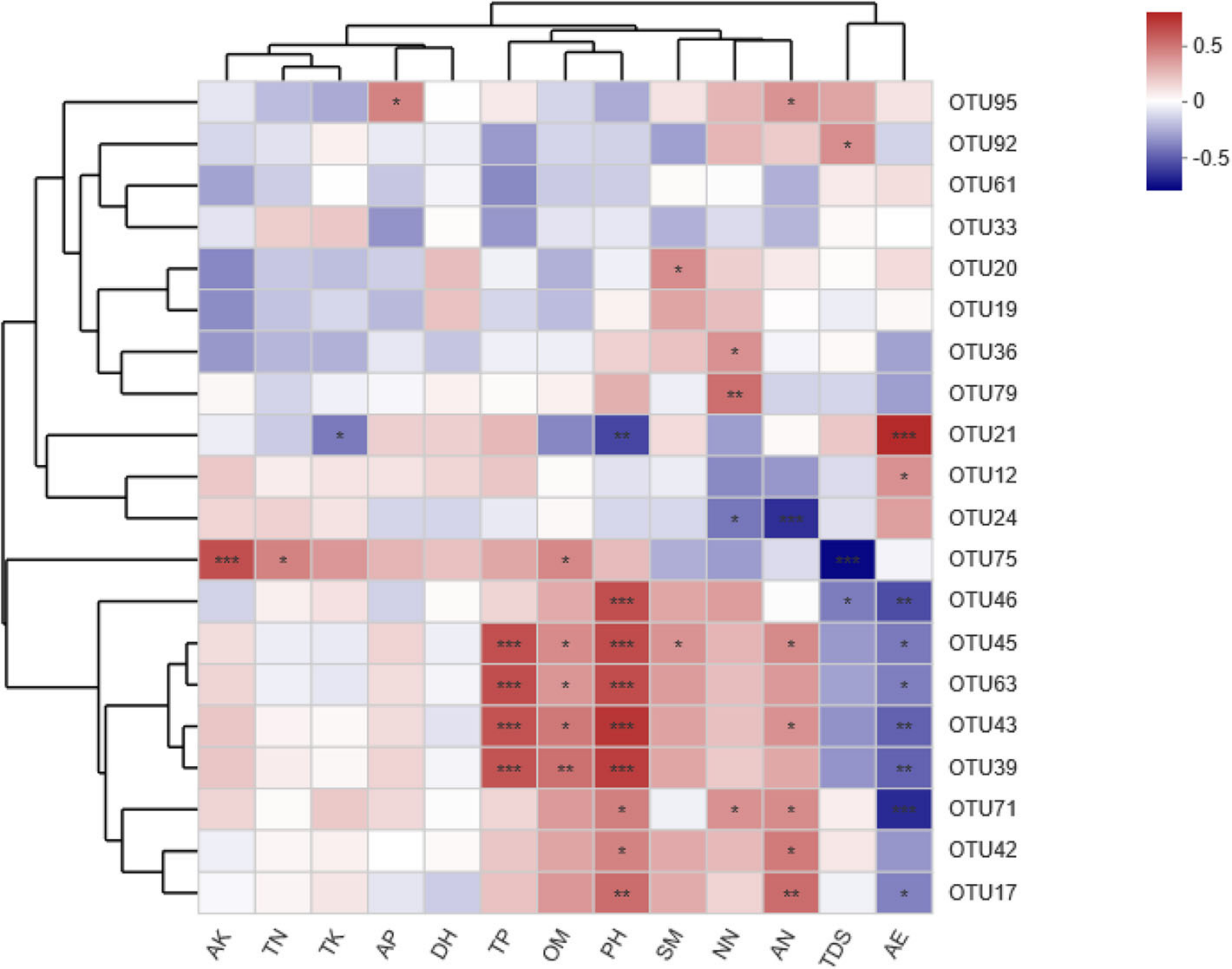
A correlation heat map of the relationship between the top 20 OTUs and soil properties. The X- and Y-axes correspond to environmental factors and OTUs, respectively. Different colors are used to indicate R values corresponding to the correlations between individual variables. *** *P* < 0.001 level, ** *P* < 0.01 level, * *P* < 0.05 level

The top 20 most abundant OTUs were identified and used to assess relationships between species and environmental factors. Among these OTUs were OTU33, which corresponded to an *Ambispora* species and OTU61, OTU12, and OTU24 which corresponded to *Diversispora* species, with all other top OTUs corresponding to *Glomus* species. OM, NN, AN, TP, AE, TDS, and pH were all significantly correlated with three or more OTUs (Fig. 6).

## Discussion

In this study, the relative diversity of *Glomus, Diversispora,* and *Ambispora* fungi varied significantly, with these genera accounting for 83.46, 14.73, and 1.21% of the fungi in analyzed samples, respectively (Fig. 2a). Owing to their adaptability, *Glomus* species are abundant in many ecosystems, consistent with our findings. As *Glomus* and *Diversispora* were the two dominant genera detected in soil samples in the present study, this suggests that these fungi are better adapted to the desert environment at this study site. In addition, we were unable to identify certain AMF species, and *Paraglomus* species were detected in only a few samples. Some molecular studies of AMF communities have reported difficulties in the detection of *Paraglomerales* and *Archaeosporales* species [35, 36], potentially due to PCR primer-related issues [37], owing to the use of different target genes or genomic regions and primer combinations that exhibit differences in specificity and efficacy across fungal genera [38, 39]. Maarten et al. demonstrated the complementary specificity of AMV4.5NF–AMDGR with AML1–AML2 primer sets, and found that a greater number of high-quality AMF sequences were obtained for the AMV4.5NF–AMDGR primers when evaluating six primer pairs targeting the nuclear rRNA operon as a means of characterizing AMF communities [38]. However, their results also suggested that this primer pair favored the amplification of *Glomeraceae* sequences at the expense of *Ambisporaceae*, *Claroideoglomeraceae,* and *Paraglomeraceae* sequences. As such, future efforts to identify more reliable primer pairs may be warranted.

Our petal chart analysis identified 12 core OTUs shared among different soil sample groups (Fig. 1), including 10 *Glomus* OTUs and 2 *Diversispora* OTUs that were present at all soil depths and slope positions. Given their universality among collected samples, we hypothesize that these fungi are closely associated with *Ferula sinkiangensis* growth, potentially suggesting that further study of these fungi may offer key insights into soil microbiology that can support artificial *Ferula sinkiangensis* cultivation.

In LEFse analyses, we determined that biomarkers [40–42] differed significantly as a function of soil depth and slope position, with decreasing biomarker levels as soil depth increased, suggesting that certain AMF species are sensitive to soil depth (Fig. 5b). We also found that most of these soil depth-sensitive AMF biomarkers were located in the lower slope position. This finding, together with the data shown in Fig. 5a, indicated that most AMF biomarkers were enriched at a soil depth of 0–20 cm in samples collected from the bottom of the slope, which may be a consequence of the fact that plant residues typically accumulate on the soil surface [33, 41, 42], particularly on relatively flat regions like those found at the bottom of a given slope. Such residues are associated with high soil nutrient contents, good ventilation, and favorable hydrothermal conditions that are conducive to the growth of soil microorganisms. Moreover, microorganisms can function synergistically with other AMF species [43, 44] to promote *Ferula sinkiangensis* growth.

Spearman correlation analyses revealed that soil physicochemical properties were significantly associated with AMF alpha diversity indices, with TP and pH being positively correlated with Shannon and Chao1 index values (*P* < 0.05). Soil phosphorus levels are one of the most important factors regulating AMF community diversity [44, 45], with certain studies having found AMF diversity to be significantly negatively correlated with AP levels [45, 46]. Herein, we found AMF diversity to be significantly positively correlated with soil TP (*P* < 0.05), whereas it was not significantly related to levels of AP. This may be due to the low levels of AP in these soil samples (1.67–6.85 mg/kg). It has been shown that the function whereby AMF species provide phosphorus to their host plants is phylogenetically conserved [47], such that different AMF phylogenetic groups would exhibit significant differences in availability. For example, the diversity of AMF communities associated with soybean roots was significantly influenced by P application [48], whereas such application did not affect AMF root colonization or the diversity/structure of AMF communities associated with tomato plants [49]. As such, it is possible that low P availability may select for functionally similar AMF species exhibiting highly efficient P uptake. In contrast, TP contents varied from 0.49–0.85 g/kg in the soil samples in the present study, suggesting a high potential phosphorus abundance in these soil samples. *Ferula sinkiangensis* growth is dependent upon the absorption of available soil phosphorus, and AMF species can facilitate such phosphorus uptake [46, 47]. This thus explains the increase in TP content, which was consistent with the substantial enrichment of AMF species adapted to low AP levels within the rhizosphere.

Soil pH is another key parameter that influences AMF community diversity, with AMF diversity often being significantly negatively correlated with pH [50]. In contrast, in the present study, we found that AMF community diversity was significantly positively correlated with soil pH, potentially due to unique local environmental factors. Some studies have shown that the tolerance of different AMF species to soil pH varies greatly [50, 51], and that the diversity and community composition of AMF species in soils with different pH values were significantly different [52–54]. The soil pH range in the present study was from 7.80–8.81, with only certain *Glomus* and *Diversispora* AMF species being able to survive in this pH range. As pH values rose, we found that the richness and diversity values corresponding to these AMFs also increased.

We detected significant differences in AMF diversity and richness as a function of slope position but not as a function of soil depth. This may be because the physical and chemical properties of soil at different slope locations differed significantly, whereas these properties did not vary as a function of soil depth. Cluster analyses (Fig. 3) clearly separated soil samples into three categories, which revealed that AMF community composition at a given slope level was similar, whereas this composition varied significantly as a function of slope level. We additionally observed no significant differences in soil properties as a function of soil depth, whereas these properties did differ at different slope positions, with significant differences being observed in OM, TP, TK, AN, pH, and SM (*P* < 0.05, Table 2). AMF diversity and richness were closely associated with environmental factors, and CCA analyses revealed that OM, TN, TK, NN, AN, AK, TDS, pH, SM, and AE all had a significant impact on AMF community composition (Fig. 4b). AE was found to be positively correlated with TDS and DE, and to be negatively correlated with other environmental factors (Fig. 4b). These factors were also correlated with AMF community composition, with OM, TN, TP, TK, AN, NN, TDS, pH, and SM all being positively correlated with the abundance of many OTUs, whereas AE and TDS were negatively correlated with the abundance of several OTUs (Fig. 6). Soil composition thus differed significantly as a function of slope position, in turn affecting AMF community diversity and richness.

## Conclusion

In summary, our results provide new insights regarding the composition and diversity of rhizosphere AMF communities associated with *Ferula sinkiangensis*. We found that *Glomus* and *Diversispora* were enriched in our samples, and that rhizosphere AMF diversity and richness varied significantly among slope positions, as evidenced by differences in *Glomus* and *Diversispora* abundance. In contrast, rhizosphere soil depth did not significantly affect overall AMF diversity, although certain AMF species were found to be sensitive to depth. In addition, the physical and chemical properties of soil varied significantly as a function of slope position (*P* < 0.05), potentially explaining the differences in AMF community composition across these slope positions. Together, we hope that our results will help guide efforts to improve soil structure and AMF communities associated with *Ferula sinkiangensis* in order to improve the protection and cultivation of this valuable medicinal plant.

## Methods

### Site description and experimental design

The chosen experimental site was located in the Yining region of Xinjiang Province, China (82°7′E, 43°44′N) [1]. We selected three 10 m × 5 m rectangular plots at the top (altitude:1121 m), middle (altitude:1087 m), and bottom (altitude:1053 m) of a slope in a region containing *Ferula sinkiangensis* plants in May 2019. We then randomly selected 9 *Ferula* plants at each of these slope positions and collected rhizosphere soil samples at depths of 0–20 cm, 20–40 cm, and 40–60 cm [55] as per the methods previously described by Riley and Barber [56, 57]. We also collected samples of the surrounding soil within 5 cm of the root system. Next, we pooled together three of the nine plants, and we similarly pooled the rhizosphere soil samples from these three plants at each depth level, as well as the surrounding soil samples. Rhizosphere soil was stored in liquid nitrogen and was sent to Shanghai Maso Bio-Pharmaceutical Techno-logy Co., Ltd. for high-throughput sequencing. Samples of surrounding soil were air-dried and were then used to assess soil physicochemical properties [58, 59]. Soil samples from the top, middle, and bottom slope positions were respectively denoted with the SW, ZW, and XW designations, while samples collected at depths of 0–20 cm, 20–40 cm, and 40–60 cm were respectively designated with the numbers 1, 2, and 3.

### Library preparation

Microbial DNA [60] was extracted from 0.5 g aliquots of each sample using the E.Z.N.A.® soil DNA Kit (Omega Bio-tek, GA, USA) according to the manufacturer’s protocols. Final DNA concentrations and purity were determined by NanoDrop 2000 UV-vis spectrophotometer (Thermo Scientific, DE, USA), and DNA quality was assessed via 1% agarose gel electrophoresis. The genomic DNA pellet was stored at − 30 °C prior to use.

The partial small subunit (SSU) region of the 18S rRNA gene was amplified via nested PCR [61, 62]. AML1F (forward primer) (5′-ATCAACTTTCGATGGTAGGATAGA-3′) and AML2R (reverse primer) (5′-GAACCCAAACACTTTGGTTTCCTTGGTTTCC-3 ‘) [38, 39] were used as the primers in the first round of amplification using a thermocycler PCR system (GeneAmp 9700, ABI, USA), whereas AMV4.5NF (forward primer) (5’-AAGCTCGTAG-TTGAATTTCG-3′) and AMDGR (reverse primer) (5′-CCCAACTATCCCTATTAATCAT-3′) [38, 61, 63] were used as the primers in the second amplification step. The first-round PCR reactions were conducted using the following thermocycler settings: 3 min at 95 °C, followed by 32 cycles of 95 °C for 30 s, 55 °C for 30 s, and 72 °C for 45 s, followed by a final extension at 72 °C for 10 min. PCR reactions were performed in triplicate, with each reaction being conducted in a 20 μL volume containing 4 μL of 5 × FastPfu Buffer, 2 μL of 2.5 mM dNTPs, 0.8 μL of each primer (5 μM), 0.4 μL of FastPfu Polymerase, and 10 ng of template DNA. After amplification, replicates from each sample were pooled and separated via 2% agarose gel electrophoresis. These first-round PCR products were diluted 10-fold and used as templates for the second-round PCR amplification step using the following thermocycler settings: 3 min at 95 °C, followed by 30 cycles of 95 °C for 30 s, 55 °C for 30 s, and 72 °C for 45 s, followed by a final extension at 72 °C for 10 min. Reaction mixtures were prepared identically to those for the first round of PCR. The resultant PCR products were purified via 2% agarose gel electrophoresis with the AxyPrep DNA Gel Extraction Kit (Axygen Biosciences, CA, USA), and DNA levels were quantified using QuantiFluor™-ST (Promega, USA) according to the manufacturer’s instructions.

### Illumina MiSeq sequencing

Purified amplicons were pooled in equimolar amounts, and paired-end sequencing (2 × 300) was conducted using an Illumina MiSeq platform (Illumina, CA, USA) according to the standard protocols produced by Majorbio Bio-Pharm Technology Co. Ltd. (Shanghai, China). Sequence read processing was performed using QIIME v.1.9.1.

### Processing of sequencing data

Raw fastq files were demultiplexed, quality-filtered using Trimmomatic, and merged via FLASH with the following criteria: (i) The reads were truncated at any site receiving an average quality score < 20 over a 50 bp sliding window. (ii) Primers were exactly matched allowing for 2 nucleotide mismatching, and reads containing ambiguous bases were removed. (iii) Sequences with > 10 bp overlap were merged based upon the overlapping sequence.

Operational taxonomic units (OTUs) were clustered with a 97% similarity cutoff using UPARSE (v.7.1 http://drive5.com/uparse/), and chimeric sequences were identified and removed using UCHIME. The taxonomy of each 18S rRNA gene sequence was analyzed using RDP Classifier (v.2.2 http://sourceforge.net/projects/rdp-classifier/) and the MaarjAM (https://www.maarjam.botany.ut.ee/) 18S rRNA database at a confidence threshold of 70%.

### Soil physicochemical properties

Soil properties were assessed as described in prior studies [59, 64]. Analyzed soil physicochemical properties included gravimetric soil water content (assessed via by oven drying at 105 °C) [65, 66], organic matter content (assessed using the KCr_2_O_7_ method) [66], total nitrogen (assessed using the HClO_4_-H_2_SO_4_ digestion method) [67, 68], total phosphorus (assessed using a Mo-Sb colorimetric method) [68, 69], total potassium (as measured via atomic absorption spectrometry) [69, 70], nitrate nitrogen, ammonium nitrogen (as assessed via a 0.01 M calcium chloride extraction method using a BRAN+LUEBBE flow analyzer) [67, 68], available phosphorus (using NaH-CO_3_ extracts and analyzed via the Mo-Sb colorimetric method) [68, 70], available potassium (measured using NH_4_OAc extracts and analyzed via atomic absorption spectrometry), pH (as measured with a Mettler Tolido FiveEasy Plus pH meter) [70], and total dissolvable salts (as assessed via atomic absorption spectrometry and titration) [59, 70].

### Data analysis

In the diversity indices, all the rarefaction and diversity analysis for community analysis was performed at the lowest number of reads (14991) per sample. The Mothur (version v.1.30.1 http://www.mothur.org/wiki/Schloss_SOP#Alpha_diversity) was used to calculate Sobs (the observed richness) as well as the Chao1 (the Chao1 estimator), Shannon (the Shannon diversity index), Simpson (the Simpson diversity index), coverage (Good’s coverage indices), and Phylogenetic diversity (PD), which were respectively used to assess sample richness, diversity, and coverage. Since the data were not normally distributed, Kruskal-Wallis test was used to detect whether there was a significant difference in index values between the groups. The dilution curve was plotted with R (v3.6.1) to count the Alpha diversity index of the corresponding samples of these sequences by randomly selecting 14,991 sequences from the samples. The extracted data volume was taken as the x-coordinate and the Alpha diversity index as the y-coordinate.

The Venn diagram diagram were drawn using R (v3.6.1) to count the number of common and unique species (such as OTU).

The community column were drawn using R (v3.6.1) to count the species composition of different groups (or samples) at each level of classification.

The Circos sample relationship diagram is a visual circle diagram describing the correspondence between samples (or groups) and species, which was drawn by Circos-0.67-7 (http://circos.ca/). The abundance of samples in the group was calculated using mean values. In all samples, species with less than 0.01 abundance were combined as others.

The Unweighted Pair-group Method with Arithmetic Means (UPGMA) clustering was performed as a type of hierarchical clustering method to interpret the distance matrix using average linkage. The UPGMA clustering based on Unweighted Unifrac Distances at the OTU level, which was conducted by QIIME (Version 1.9.1) and drawn by R (v3.6.1).

Principal co-ordinate analysis (PCoA) based on bray-curtis at OUT level analysis the community of AMF by R (v3.6.1). The Analysis of similarities (ANOSIM) in “vegan” R package is used to examine differences between groups. The test for significance is 999 permutations.

Differences between groups were assessed based upon linear discriminant analysis (LDA) effect size (LEfSe) (http://huttenhower.sph.harvard.edu/galaxy/root?tool_id=lefse_upload). First, the non-parametric factorial Kruskal-Wallis (KW) sum-rank test is applied to detect the significant difference of abundance and find out the group with significant difference. Finally, linear discriminant analysis (LDA) estimate the impact of abundance of each component (species) on the difference effect, the microbes of LDA (> 2) are considered to be significant groups of microbes. The multi-group comparison strategy is that species can only be considered as differential species if there are differences in multiple groups.

Since the axis length > 4 in the DCA results, canonical correlation analysis (CCA) was used to test the relationship among environmental factors, samples and microbes. The CCA was estimated using the “vegan” package in R (v3.6.1).

Correlations between soil properties and dominant OTUs were assessed via Spearman correlation analyses, while relationships between rhizosphere composition, soil properties. The Correlation Heatmap was drawn using the “pheatmap” package in R (v3.6.1).

Since the data were not normally distributed, Kruskal-Wallis test was used to detect whether there was a significant difference in the soil physicochemical factors between the groups. Spearman correlation analyses were also run among the soil physicochemical factors with Alpha diversity index values. The Statistical analysis was carried out with SPSS 19.0 (IBM Inc., Armonk, USA).

## Supplementary Information

**Additional file 1: Table S1.** The relationships between alpha diversity indices and abiotic factors. Spearman correlation analyses of the relationships between alpha diversity indices and abiotic factors in all samples. Only variables with significant relationships are shown. ** *P* < 0.01 level (two-tailed). * *P* < 0.05 level (two-tailed). **Figure S1.** Rhizosphere soil sample dilution curves. SW, ZW, and XW respectively correspond to the top, middle, and bottom slope positions. 1, 2, and 3 respectively represent samples collected at a soil depth of 0-20 cm, 20-40 cm, and 40-60 cm. **Figure S2.** A Circos sample relationship diagram. The small left semi-circle corresponds to the species composition within a given sample, while the color of the outer ribbon corresponds to the group, the color of the inner ribbon corresponds to the species, and the length of the ribbons correspond to the relative abundance of these species within the indicated sample. The right semi-circle corresponds to the distributions of species within different samples at the taxonomic level, the outer band represents species, the inner band represents different groups, and the length corresponds to the distribution proportion of the sample in a given species. SW, ZW, and XW respectively correspond to the top, middle, and bottom slope positions. 1, 2, and 3 respectively represent samples collected at a soil depth of 0-20 cm, 20-40 cm, and 40-60 cm.

## Data Availability

The datasets generated and/or analysed during the current study are not publicly available due the datasets also forms part of an ongoing study, but they are available from the corresponding author on reasonable request.

## Abbreviations

OM: Organic matter content
TN: Total nitrogen
TP: Total phosphorus
TK: Total potassium
NN: Nitrate nitrogen
AN: Ammonium nitrogen
AP: Available phosphorus
AK: Available potassium
TDS: Total dissolvable salt
pH: Hydrogen ion concentration
SM: Soil water content
AE: Slope position
DE: Depth
