# Supplementary material for: High-throughput sequencing analysis of the rhizosphere arbuscular mycorrhizal fungi (AMF) community composition associated with Ferula sinkiangensis
Source: BMC Microbiol. 2020 Nov 3;20:335. doi: 10.1186/s12866-020-02024-x (PMC7640387; doi:10.1186/s12866-020-02024-x)
Supplement: Supplementary file 1 — Additional file 1: Table S1. The relationships between alpha diversity indices and abiotic factors. Spearman correlation analyses of the relationships between alpha diversity indices and abiotic factors in all samples. Only variables with significant relationships are shown. ** P < 0.01 level (two-tailed). * P < 0.05 level (two-tailed). Figure S1. Rhizosphere soil sample dilution curves. SW, ZW, and XW respectively correspond to the top, middle, and bottom slope positions. 1, 2, and 3 respectively represent samples collected at a soil depth of 0-20 cm, 20-40 cm, and 40-60 cm. Figure S2. A Circos sample relationship diagram. The small left semi-circle corresponds to the species composition within a given sample, while the color of the outer ribbon corresponds to the group, the color of the inner ribbon corresponds to the species, and the length of the ribbons correspond to the relative abundance of these species within the indicated sample. The right semi-circle corresponds to the distributions of species within different samples at the taxonomic level, the outer band represents species, the inner band represents different groups, and the length corresponds to the distribution proportion of the sample in a given species. SW, ZW, and XW respectively correspond to the top, middle, and bottom slope positions. 1, 2, and 3 respectively represent samples collected at a soil depth of 0-20 cm, 20-40 cm, and 40-60 cm. [file 12866_2020_2024_MOESM1_ESM.pdf]

Supplementary Figure S1. Rhizosphere soil sample dilution curves.

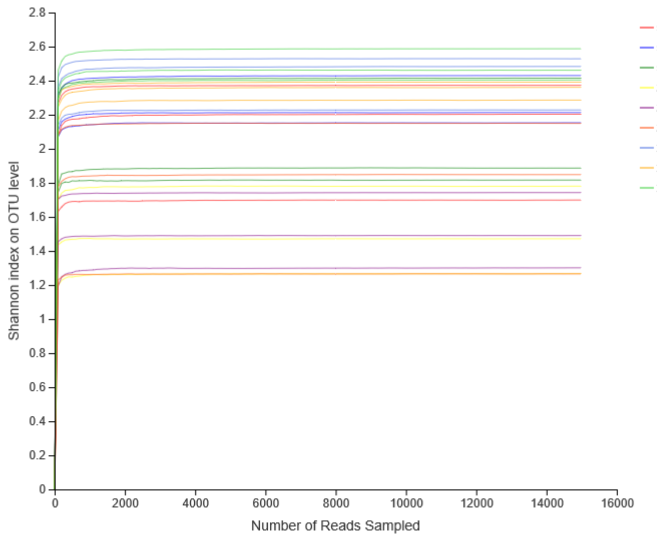

SW, ZW, and XW respectively correspond to the top, middle, and bottom slope positions. 1, 2, and 3 respectively represent samples collected at a soil depth of 0-20cm, 20-40cm, and 40-60cm.

Supplementary Figure S2. A Circos sample relationship diagram.

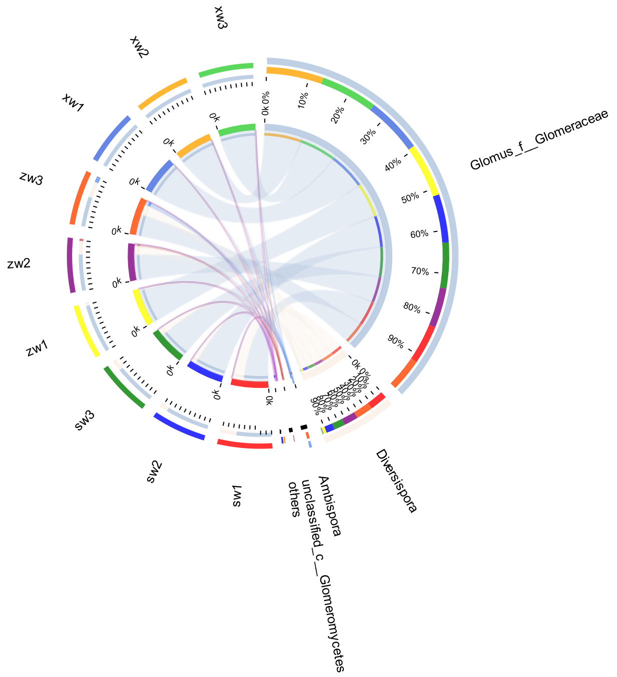

The small left semi-circle corresponds to the species composition within a given sample, while

the color of the outer ribbon corresponds to the group, the color of the inner ribbon corresponds to the species, and the length of the ribbons correspond to the relative abundance of these species within the indicated sample. The right semi-circle corresponds to the distributions of species within different samples at the taxonomic level, the outer band represents species, the inner band represents different groups, and the length corresponds to the distribution proportion of the sample in a given species. SW, ZW, and XW respectively correspond to the top, middle, and bottom slope positions. 1, 2, and 3 respectively represent samples collected at a soil depth of 0-20cm, 20-40cm, and 40-60cm.

Supplementary Table

Supplementary Table S1. The relationships between alpha diversity indices and abiotic factors.

	TP	AN	NN	TDS	PH	AE
Shannon	0.521**	0.325	0.522**	-0.109	0.496**	-0.361
Chao **1**	0.504**	0.332	0.204	-0.501**	0.562*	-0.414*

Spearman correlation analyses of the relationships between alpha diversity indices and abiotic factors in all samples. Only variables with significant relationships are shown. ** P<0.01 level (two-tailed). * P<0.05 level (two-tailed)
